# Synthesis of Aroma Compounds as a Function of Different Nitrogen Sources in Fermentations Using Non-*Saccharomyces* Wine Yeasts

**DOI:** 10.3390/microorganisms11010014

**Published:** 2022-12-21

**Authors:** Jennifer Badura, Marko Medić, Niël van Wyk, Birgit Krause, Heike Semmler, Silvia Brezina, Isak S. Pretorius, Doris Rauhut, Christian von Wallbrunn

**Affiliations:** 1 Department of Microbiology and Biochemistry, Hochschule Geisenheim University, Von-Lade-Strasse 1, 65366 Geisenheim, Germany; 2 ARC Centre of Excellence in Synthetic Biology, Department of Molecular Sciences, Macquarie University, Sydney, NSW 2113, Australia; 3 Department of Soil Science and Plant Nutrition, Hochschule Geisenheim University, Von-Lade-Strasse 1, 65366 Geisenheim, Germany

**Keywords:** fermentation, amino acids, nitrogen consumption, aroma profile, *Hanseniaspora*, *Zygosaccharomyces*, *Starmerella*

## Abstract

Non-*Saccharomyces* yeasts are prevalent at the onset of grape must fermentations and can have a significant influence on the final wine product. In contrast to *Saccharomyces cerevisiae*, the biosynthetic pathways leading to aroma compound formation in these non-conventional yeasts, in particular those that are derived from amino acid metabolism, remains largely unexplored. Within a synthetic must environment, we investigated the amino acid utilization of four species (*Hanseniaspora uvarum*, *Hanseniaspora osmophila*, *Zygosaccharomyces rouxii*, *Starmerella bacillaris*) and *S. cerevisiae*. We report on the differential uptake preferences for amino acids with *H. uvarum* displaying the most rapid uptake of most amino acids. To investigate the fate of amino acids and their direct contribution to aroma synthesis in *H. uvarum*, *H. osmophila* and *Z. rouxii*, musts were supplemented with single amino acids. Aroma profiling undertaken after three days showed the synthesis of specific aroma compounds by the respective yeast was dependent on the specific amino acid supplementation. *H. osmophila* showed similarities to *S. cerevisiae* in both amino acid uptake and the synthesis of aroma compounds depending on the nitrogen sources. This study shows how the uptake of specific amino acids contributes to the synthesis of aroma compounds in wine fermentations using different non-*Saccharomyces* yeasts.

## 1. Introduction

Managing the nitrogen content in grape must is essential to prevent stuck or sluggish fermentations [1]. The forms of nitrogen utilized by the microbiota within grape must are collectively known as the yeast assimilable nitrogen (YAN) and consist of ammonium ions (NH_4_^+^) and free amino nitrogen (FAN). FAN is a combination of individual amino acids and small peptides and most notably excludes the abundant amino acid L-proline as it cannot be utilized by yeast under anaerobic conditions. Insufficient starting levels of YAN (below 100 mg/L for red wines and 150 mg/L for white wines) can be remedied by the addition of inorganic forms of nitrogen like diammonium phosphate (DAP) or even more complex protein supplementations. Increased levels of YAN in must directly contribute to greater fermentation vigour, yet often lead to increased microbial instability, increased haze formation, increased biogenic amine levels, and could also contribute to atypical aging properties [2]. It is thus pivotal to provide the optimal amount of YAN to ensure a successful fermentation without causing any type of wine faults.

**Figure 1:**
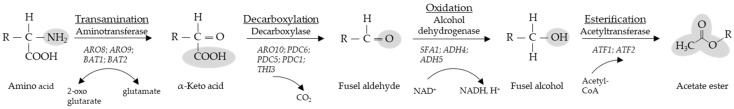
The Ehrlich pathway. Conversion of amino acids into fusel aldehydes, fusel alcohols and acetate esters. Enzymes and encoding genes known in *S. cerevisiae* to catalyze the reactions are indicated. Specific molecular groups are highlighted. Modified from Hazelwood et al. [10].

Most of our understanding of how nitrogen is consumed during wine fermentation is obtained from studies conducted with *S. cerevisiae*, which is mainly responsible for the alcoholic fermentation. Nitrogen utilization is a highly regulated process and at least four different regulatory mechanisms have been described: the Ssy1-Ptr3-Ssy5 system (SPS), the nitrogen catabolic repression (NCR), the retrograde signalling pathway (RTG) and the general amino acids control (GAAC) [3]. All the above-mentioned mechanisms are in turn governed by the target of rapamycin (TORC1) signalling pathway. Several allelic variants in key genes involved in the TORC1 signalling pathway have been identified to explain possible adaptations of wine strains of *S. cerevisiae* and their consumption preference for specific amino acids during wine fermentation [4]. Nitrogen, both form and quantity, also has a major impact on the aroma profile and thus the sensorial characteristics of the final wine product [5,6]. The breakdown of amino acids via the Ehrlich pathway results in the production of higher alcohols like isoamyl alcohol and 2-phenylethyl alcohol which are some of the key aroma determinants in wine along with their corresponding acetate esters (Figure 1, Table 1) [7]. With regards to wine aroma, insufficient YAN has also been implicated in excessive H_2_S production, yet excessive YAN has been shown to lead to increased volatile acidity and increased ethyl acetate production [8,9]. 

At the onset of a grape must fermentation, the microbial population structure is particularly heterogeneous consisting of numerous yeast genera. It is often the case that only a small proportion of the population is comprised of *Saccharomyces* spp. [11]. This is why commercial winemaking largely employs starter cultures of *S. cerevisiae*, as this ensures early onset of fermentation, successful conversion of the grape must sugars, and reproducible fermentation results. With spontaneous fermentations, when no starter culture is added, the *S. cerevisiae* population tends to not just increase, but manages to suppress the non-*Saccharomyces* yeast (NSY) populations. Yet, spontaneous fermentations are at a far greater risk of becoming stuck due to the initial competition for nutrients among the different yeast species. Nevertheless, many of the NSY have enjoyed increasing popularity in recent years as co-partners with *S. cerevisiae* wine yeasts in so-called mixed-culture fermentations [12]. Their deliberate additions provide many beneficial attributes ranging from lower alcohol levels to more complex aroma profiles [13] and currently several different NSY starter cultures have become commercially available, e.g., *Torulaspora delbrueckii* and *Pichia kluyveri* [14]. 

It is important not to study *S. cerevisiae* in isolation but to consider the NSY populations and their roles in nitrogen consumption during winemaking. A major uptick in studies on this topic has occurred over the past couple of years, where the nitrogen metabolism of key NSYs has been investigated. Studies ranged from the nitrogen preference of NSYs under winemaking conditions to comparing their nitrogen regulation mechanisms to that of *S. cerevisiae* [15,16,17]. The amino acid preferences can vary significantly depending on many environmental parameters [18]. For *S. cerevisiae*, diverse amino acid preferences have been reported in fermentations of grape must and beer wort [19,20]. For example, it was found that, in grape must fermentations, Lys was the most preferred amino acid to be utilized, followed by early consumption of Asp, Thr, Glu, Leu, His, Met, Ile, Ser, Gln, and Phe [19]. The amino acids Val, Arg, Ala, Trp, and Tyr were consumed slower. In beer wort, the most preferred amino acids that were the first to disappear in the medium were Glu, Asp, Asn, Gln, Ser, Thr, Lys and Arg, followed by the branched-chain amino acids Ile, Leu and Val as well as Met and His [20]. The amino acids Gly, Ala and the aromatic amino acids Phe, Tyr and Trp were detected longer in the fermentation supernatant and disappeared before the amino acid Pro.

**Table 1:** Ehrlich pathway intermediates and derivates of branched-chain amino acids (Leu, Val, Ile), aromatic amino acids (Phe, Tyr, Trp), the sulfur-containing amino acids (Met, Cys) and the polar-uncharged amino acid (Thr) as well as the odor descriptors of their acetate esters. Modified from Dzialo et al. [7], Valera et al. [21] and Vermeulen et al. [22].

Amino Acid	α-Keto Acid	Fusel Aldehyde	Fusel Alcohol	Acetate Ester	Odor Descriptor of Acetate Ester
Leu	α-Keto-isocaproate	3-Methyl- butanal	Isoamyl alcohol	Isoamyl acetate	Banana, sweet, fruity
Val	α-Keto-isovalerate	2-Methyl- propanal	Isobutanol	Isobutyl acetate	Sweet, fruity, tropical
Ile	α-Keto-3-methylvalerate	2-Methyl- butanal	2-Methylbutanol	2-Methylbutyl acetate	Fruity, tropical, overripe fruit
Phe	Phenylpyruvate	2-Phenyl- ethanal	2-Phenyl ethanol	2-Phenylethyl acetate	Floral, rose, honey
Tyr	4-Hydroxyphenylpyruvate	2-(4-Hydroxyphenyl) ethanal	Tyrosol	Tyrosol acetate	Fruity, flowery
Trp	Indol-3-pyruvate	2-(Indol-3-yl) ethanal	Tryptophol	Tryptophol acetate	Fruity, flowery
Met	2-Keto-4-methylthio-2-oxobutyrate	3-(Methylthio) propanal	Methionol	Methionyl acetate	Cabbage, potato, mushroom
Thr	α-Keto-butyrate	Butanal	Butanol	Butyl acetate	Solvent, fruity, banana
Cys	3-Mercapto- pyruvate	Mercaptoacetaldehyde	2-Mercaptoethanol	2-Mercaptoethyl acetate	Roasted

In this study, we determined amino acid consumption profiles for one *S. cerevisiae* commercial wine strain (Geisenheim/Uvaferm GHM) and the non-*Saccharomyces* yeast species *Hanseniaspora uvarum*, *H. osmophila*, *Zygosaccharomyces rouxii* and two strains of *Starmerella bacillaris*, that can be found at the onset of grape must fermentation. Subsequently, we carried out fermentations with three of the non-*Saccharomyces* yeast strains (*H. uvarum*, *H. osmophila* and *Z. rouxii*) in synthetic musts that were spiked with one amino acid as an additional nitrogen source in order to determine the amino acid-dependent production of aroma compounds.

## 2. Materials and Methods

### 2.1. Yeast Strains and Media

The following yeast strains were used: the commercial *S. cerevisiae* wine yeast strain Geisenheim/Uvaferm GHM (Lallemand, Montreal, Canada), *H. uvarum* DSM2768, *H. osmophila* NRRL Y-1613^T^, *S. bacillaris* GYBC-240 and GYBC-241 as well as the *Z. rouxii* GYBC-242. The latter three strains were obtained from the Geisenheim Yeast Breeding Center culture collection.

Yeasts were routinely cultured in YPD medium (20 g/L glucose, 20 g/L bacto peptone and 10 g/L yeast extract). For the preparation as preculture for fermentation experiments, yeast strains were grown in synthetically defined (SD) media (20 g/L glucose, 0.17 g/L yeast nitrogen base and 0.23 g/L ammonium chloride [16]) and washed in a sterilized 9 g/L NaCl solution.

The synthetic grape must (SM) medium was prepared according to Bely et al. [23], with adjustments from Su et al. [24] and Seguinot et al. [25]. The composition of the synthetic grape must, without the addition of the amino acids (AA), can be found in Table 2. 

The pH of the SM medium was adjusted to pH 3.3 using 5N NaOH. The nitrogen content was 140 mg/L of YAN. For experiments using an amino acid mixture (SM-mix), amounts of amino acids resembling the natural composition found in grapes were added, with 70% of the nitrogen coming from amino acids and 30% from ammonium chloride. For experiments with single amino acid fermentations (SM-AA), amino acids were added in amounts derived from their molecular mass and number of assimilable nitrogen atoms in the molecule. The concentrations of amino acids used were taken from Su et al. [16]. The concentrations of the added amino acids for the fermentations with the amino acid mixture and the addition of individual amino acids are listed in Table 3. All amino acids used were purchased either from Merck (Darmstadt, Germany) or Carl Roth (Karlsruhe, Germany).

### 2.2. Fermentation Experiments

We followed three experimental lines in our fermentation experiments. On the one hand, fermentation kinetics of all individual yeast strains in SM-mix were determined using the ANKOM Rf Gas Production system (ANKOM Gesellschaft für Analysentechnik—HLS, Salzwedel, Germany) as described previously [26]. Therefore, 150 mL of SM-mix were transferred into a modified 250 mL borosilicate bottle sealed with a lid containing the Rf sensor module of the ANKOM Rf Gas Production system and inoculated with 1 × 10^6^ cells/mL. The bottles were equipped with appropriate magnetic stirrer bars before placing on magnetic stirrer pads, enabling constant stirring. The interval for measuring the prevailing pressure was set to 30 min. The pressure was measured for 237.5 h (~10 days).

Secondly, the rate at which each amino acid was consumed within a mixture of amino acids was evaluated. All six yeast strains were used to ferment the 150 mL SM-mix (in 300 mL Erlenmeyer flasks) with the nitrogen content as described in Table 2 and Table 3. The yeast pitching rate was 1 × 10^6^ cells/mL. Appropriate dilutions of starting cultures were prepared according to cell numbers determined by haemocytometer cell counts. Flasks were covered with aluminium foil. Fermentations were conducted in a temperature-controlled shaking incubator at 25 °C and shaken at 120 min^−1^. Samples (2 mL) were removed every 8 h for the first 32 h, pelleted by centrifugation at 13.000 min^−1^ for 1 min and stored at −80 °C for later analysis of the amino acid composition via ion exchange chromatography (IEC). After 72 h, samples were also prepared for aroma compound analysis via gas chromatography/mass spectrometry (GC/MS) and quantification of organic acids, ethanol, and sugar concentrations via high-performance liquid chromatography (HPLC).

The last line of experiments evaluated the aroma compound formation of must fermentations that were enriched with individual amino acids. The strains *H. uvarum* DSM2768, *H. osmophila* NRRL Y-1613^T^ and *Z. rouxii* GYBC-242 were used in this experiment. High-throughput fermentations were carried out at 25 °C in 35 mL of SM-AA (see Table 3) in 50 mL conical fermentation tubes fitted in tube racks to allow stirring at 120 min^−1^. The nitrogen content of SM-AA totalled 140 mg/L of YAN. Tubes were sealed with aluminium foil. Fermentations were carried out for 72 h. Samples were analysed via GC/MS and HPLC.

### 2.3. Analysis of Amino Acid Uptake with Ion Exchange Chromatography (IEC)

Concentrations of amino acids were undertaken on an ARACUS Amino Acid Analyzer Advanced (membraPure GmbH, Hennigsdorf, Germany). The analyser uses post-column derivatisation with ninhydrin and cation exchange chromatography [27]. Samples were prepared by mixing 600 μL of sample buffer (lithium-citrate solution 0.12 M, pH 2.2, 26.23 µg/mL norleucine as internal standard; Sykam, Eresing, Germany) with 600 μL of the sample. Samples were passed through a nylon filter (syringe filter, nylon, 0.45 μm, MS Scientific, Berlin, Germany) into glass vials to analyse free amino acids. Prior to photometric detection of primary and secondary amino acids via UV/VIS-detector at 570 nm and 440 nm, respectively, automatic post-column ninhydrin derivatisation at 130 °C, leading to a colourisation of the amino acids, was performed for quantification according to Krause and Löhnertz [28]. This treatment dyes primary amino acids blue-violet and secondary amino acids (i.e., Pro) yellow. Samples up to and including the 32 h timepoint were analysed for each strain. 

Since cysteine is not stable in solution and oxidizes to form cystine, cysteine could not be detected in the fermentation supernatant. Due to the deamination of glutamine to glutamic acid and asparagine to aspartic acid, neither glutamine nor asparagine could be measured in the fermentation supernatants. Accordingly, the glutamic acid values contain the sum of glutamine and glutamic acid at the respective time of sampling. The same applies to the values of aspartic acid and asparagine.

### 2.4. High-Performance Liquid Chromatography (HPLC) Analysis

Sugars (glucose and fructose), organic acids (tartaric acid, malic acid, lactic acid, acetic acid and citric acid) and ethanol were measured by HPLC using a method as described previously [29,30]. An Agilent Series 1100 (Agilent Technologies Inc., Santa Clara, CA, USA) HPLC with UV/VIS (at 210 nm) and Refractive Index (35 °C) detectors and an Allure Organic Acids 5 μm particle size (length 250 mm, diameter 4.6 mm; Restek GmbH, Bad Homburg vor der Höhe, Germany) column was used. As eluent, a 0.5% ethanol/ 0.0139% H_2_SO_4_ solution was used at a flowrate of 0.5 mL/min at 46 °C. Quantification was aided by the use of external standards. For sample preparation, thawed samples were centrifuged at 13,000 min^−1^ for 10 min and the supernatant subsequently diluted fourfold with ultrapure water along with the addition of 55 μL of 10% ethanol.

### 2.5. Analysis of VOCs with Head-Space Solid Phase Microextraction Gas Chromatography (HS-SPME-GC-MS)

The concentrations of volatile aroma compounds were measured by head-space (HS) solid phase microextraction (SPME) GC/MS (HS-SPME-GC-MS) as previously described [31,32]. A 7890 A gas chromatograph with a 5975-B mass spectrometer (both Agilent Technologies Inc., USA) was utilised. SPME was undertaken with a 1 cm length and 65 μm particle size polydimethylsiloxane and divinylbenzol fiber (Merck, Germany) at 40 °C and 10 min incubation time. Extraction was performed at 500 min^−1^ for 20 min. For the GC a Rxi^®^-5Si1 MS (Restek GmbH, Germany) column of 60 m length, 0.25 mm diameter and 1 μm particle size was used with helium as the carrier gas. Sample injection was performed 1:10 split mode and heating from 30 °C to 240 °C with a 12 °C/s raise and a 4 min hold. The GC run started with an initial 4 min hold at 40 °C after which the temperature was raised to 210 °C at a 5 °C/ min rate and to 240 °C with 20 °C/ min and a final 10.5 min hold. Data from the mass spectrometer used for the concentration values were in the range of 35 to 250 of the m/z masses to charge ratio. For the calibration of each measured compound a 5-point curve was made with a 3%, 6%, 9% or 12% ethanol, respectively, and 3% tartaric acid solution at pH 3, depending on the ethanol content of the samples. Samples were prepared by adding 1.7 g NaCl to 5 mL of sample in a 20 mL brown head-space glass vials. Two internal standard solutions (10 μL) were also added, namely 600 mg/L 1-octanol and 52 mg/L cumene.

### 2.6. Statistical Evaluation and Software

All fermentation experiments were performed in triplicates. For the statistical evaluation two-tailed unpaired *t*-test with Welch’s correction was used to analyse the data in GraphPad PRISM software (Version 9.4.1) and significance was set at *p* < 0.05 to the control (GraphPad Software, San Diego, CA, USA). HPLC data were analysed using the software ChemStation for LC systems from Agilent (Agilent, Santa Clara, CA, USA). Agilent’s MassHunter software (Agilent, Santa Clara, CA, USA) was used for analysing the HS-SPME-GC-MS data. IEC data were analysed by Clarity chromatography software (DataApex, Prague, Czech Republic).

## 3. Results

### 3.1. Fermentation Kinetics

**Figure 2:**
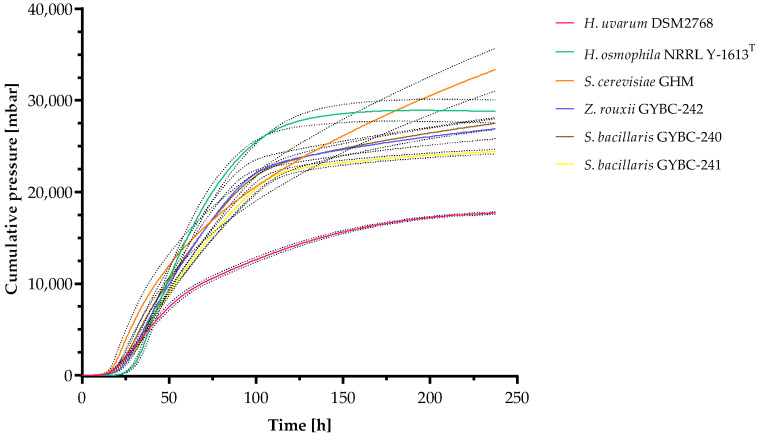
Fermentation kinetics of fermentations with SM-mix. Cumulative pressure was measured using ANKOM Rf Gas Production system. Data are the mean of three independent experiments ± SEM. Dashed lines indicate the standard deviation.

Fermentations of all individual yeast strains in SM-mix were performed to analyze the fermentation kinetics and evaluate their fermentation performance within this must (Figure 2). The cumulative pressure of fermentations using *H. osmophila* reached a plateau phase after ~180 h (~7.5 days) indicating the end of fermentation. Fermentations with the other yeast strains showed slightly increasing pressure until the end of the experiment, with *S. cerevisiae* having a steadier increase reaching the highest value of cumulative pressure measured. In contrast, the fermentations with *H. uvarum* yielded the lowest value of cumulative pressure, which was about half of the pressure achieved by *S. cerevisiae*. Fermentations using both *Z. rouxii* and the *S. bacillaris* strains showed similar patterns in fermentation performance, slightly resembling in appearance to that of *H. osmophila*. Among all yeasts, *S. cerevisiae* was the first one reaching a cumulative pressure of one bar (~18.75 h) in the SM-mix fermentations, followed by S. bacillaris GYBC-240 (~20.67 h) and H. uvarum (~21 h). *Z. rouxii* and *S. bacillaris* GYBC-241 (both ~23.67) were found to exceed this value even before *H. osmophila* (~29.67 h).

### 3.2. Evaluation of Amino Acid Consumption Rate

**Figure 3:**
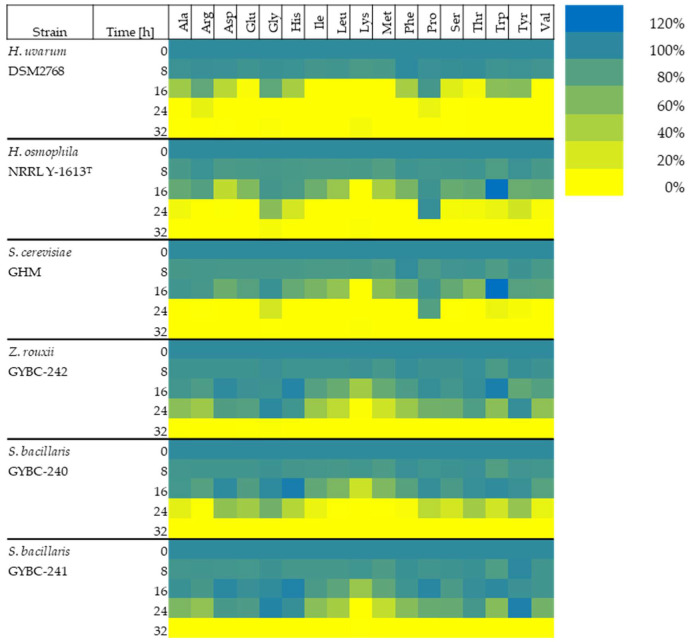
Amino acid concentration (%) present in the medium at different time points of the alcoholic fermentation in SM-mix. The initial concentration of each amino acid is expressed as 100%. It should be noted that the values of Glu also contained Gln and Asp also contained Asn. Cys could not be measured.

To evaluate the consumption rate of 17 amino acids (Cys, Asn and Gln could not be measured), we generated a synthetic must supplemented with defined amounts of these amino acids (Table 2). Fermentations of these musts by *H. uvarum*, *H. osmophila*, *Z. rouxii* GYBC-242 and *S. bacillaris* GYBC-240 & GYBC-241 were carried out over a duration of 32 h (Figure 3, Figure S1) and the amino acid concentrations in the supernatants of these fermentations were quantified in eight-hour intervals. The results of the amino acid uptake of the yeasts at the individual time points compared to the initial values are described below.

Eight hours after inoculation, the concentrations of the individual amino acids in the fermentation supernatant ranged from 83% to 100% for all yeast cultures. In the fermentations with *H. uvarum*, Lys (87%) and Met (88%) were present in the lowest concentrations. In both the fermentations with *H. osmophila* and *S. cerevisiae*, the lowest concentrations of amino acids were Met (*H. osmophila*: 83%, *S. cerevisiae*: 84%), followed by Trp (both 85%). The concentration of Trp had decreased the most after eight hours in the fermentations with *Z. rouxii* (87%) and both *S. bacillaris* strains (GYBC-240: 83%, GYBC-241: 85%), followed by Met (*Z. rouxii* GYBC-242: 89%, *S. bacillaris* GYBC-240 & GYBC-241: 86%).

Sixteen hours after inoculation, the branched-chain amino acids (Ile, Leu and Val) as well as Lys and Met were completely depleted in the fermentations with *H. uvarum*, while the concentrations of Glu and Thr were below 6%. Additionally, the concentrations of Ala, Asp, His, Phe and Ser diminished to 15–50%. Arg, Gly, Pro, Trp and Tyr were still present at more than 50%. In the fermentations with the other yeasts, however, the amino acids were present in much higher concentrations at 16 h. Only Lys was completely utilized in the fermentations with *H. osmophila* and also *S. cerevisiae* after 16 h. The concentrations of the other amino acids in these cultures ranged from 30% to 95%. An exception was Trp, which was present in both cultures at higher concentrations than the initial concentration at this time (120%). In the fermentations with both *Z. rouxii* and the two *S. bacillaris* strains, it was also Lys that was present in the lowest concentration 16 h after the start of fermentation (*Z. rouxii* GYBC-242: 46%, *S. bacillaris* GYBC-240: 25%, *S. bacillaris* GYBC-241: 50%). The concentrations of the other amino acids in these cultures ranged from 60% to 100%. Histidine was present in all three cultures at slightly higher than initial concentrations at that time (105–111%). The concentration of Trp was also increased in the cultures with *Z. rouxii* (110%), as with the cultures of *H. osmophila* and *S. cerevisiae*. Twenty-four hours after inoculation, almost all amino acids were consumed in the cultures with *H. uvarum*. Only 12% of Arg and 10% of Pro were still present. In the fermentations with *H. osmophila* and *S. cerevisiae*, on the other hand, a Pro concentration of 97% and 83%, respectively, was still measured after 24 h. Furthermore, the following amino acids were detected at this time in *H. osmophila*: Ala, Gly, His, Ser, Thr, Trp and Tyr (see also Figure S1). Glycine, Trp and Tyr were also measured in fermentations with *S. cerevisiae* 24 h after inoculation. The concentrations of the measured amino acids in the fermentations with *Z. rouxii* and the two *S. bacillaris* strains at the 24 h time point, ranged from completely depleted to unconsumed. After 24 h, the concentration of Lys was below 2% in all three cultures. In the fermentations with *S. bacillaris* GYBC-240, only very low amounts of Arg, Leu, Met and Phe were detected (1–3%). Thirty-two hours after the start of fermentation, all amino acids were used up in all yeast cultures.

### 3.3. Analysis of Sugar Content

To evaluate the sugar consumption of the six different yeast strains, the sugar concentrations were determined via HPLC analysis and are listed in Table 4. In the SM-mix fermentations with 200 g/L starting sugar concentration, those with *H. osmophila* and *S. cerevisiae* showed a residual sugar concentration below 100 g/L, while *H. uvarum*, *Z. rouxii* and the two *S. bacillaris* strains contained sugar levels above 100 g/L. As glucose and fructose were initially present in equal amounts, the rate of assimilation of these sugars is of interest. Comparing glucose to fructose, the glucose concentrations in the fermentation supernatants of *S. cerevisiae* and both *Hanseniaspora* species were lower than the fructose concentrations. In contrast, glucose was barely utilized in the fermentation supernatant in the fermentations with *Z. rouxii* and both *S. bacillaris* strains. Looking at the ratio of glucose to fructose, apart from the original must (SM), it was most balanced in the *H. uvarum* fermentations.

Ethanol production and formation of acetic acid during fermentation with individually supplemented amino acids are given in Figure 3.

### 3.4. Volatile Organic Compounds Produced during Single Amino Acid Fermentations

**Figure 4:**
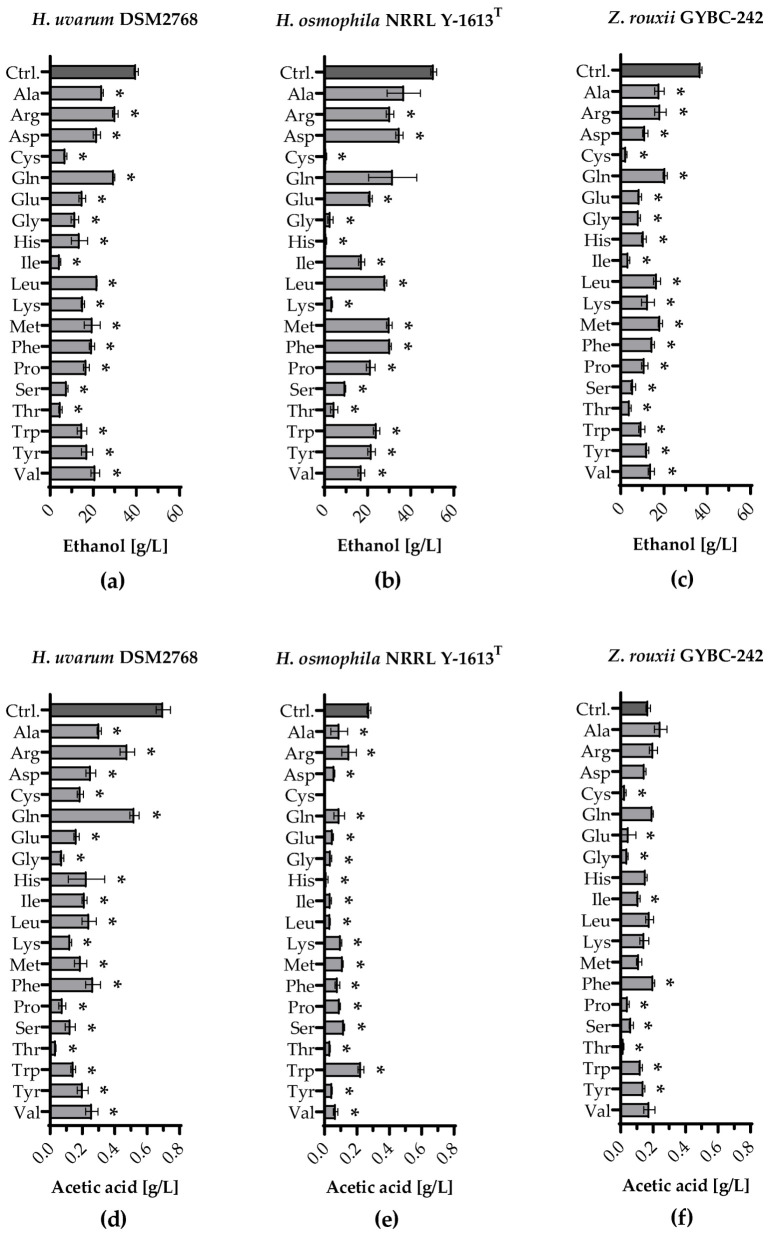
Ethanol production and formation of acetic acid during fermentation with individually supplemented amino acids. Ethanol production [g/L] in fermentations using (**a**) *H. uvarum* DSM2768, (**b**) *H. osmophila* NRRL Y-1613^T^ and (**c**) *Z. rouxii* GYBC-242. Formation of acetic acid [g/L] in fermentations using (**d**) *H. uvarum* DSM2768, (**e**) *H. osmophila* NRRL Y-1613^T^ and (**f**) *Z. rouxii* GYBC-242. Fermentations using the respective yeast with SM-mix served as control (Ctrl.). Ethanol and acetic acid concentrations were measured via high performance liquid chromatography (HPLC) analysis. Data are the mean of three independent experiments ± SEM, two-tailed unpaired *t* test with Welch’s correction, * *p* < 0.05 as compared to the control. Error bars indicate the standard deviation.

Fermentations using *H. uvarum*, *H. osmophila* and *Z. rouxii* in SM were spiked with single amino acids (SM-AA) to analyse their impact on volatile aroma compound synthesis. A fermentation with the respective yeast with the addition of an amino acid mix served as a control (Ctrl.). Figure 4 shows the amounts of ethanol and acetic acid formed. SM-mix fermentations with yeasts achieved the highest concentration of ethanol. Except for SM-AA fermentations with *H. osmophila* with Ala and Gln, significantly less ethanol was measured in the SM-AA fermentations with all three yeast strains. *H. uvarum* produced the least ethanol when Cys (~7 g/L), Ile (~5 g/L) or Thr (~5 g/L) were added and the most when Arg or Gln (both ~30 g/L) were added [Figure 4a]. The lowest ethanol concentrations were also found in fermentations using *Z. rouxii* when Cys (~3 g/L), Ile (~4 g/L) or Thr (~4 g/L) were added. The highest ethanol concentration was measured when Gln (~21 g/L) was added [Figure 4c]. Fermentations with *H. osmophila* showed the lowest ethanol concentrations when Cys (~1 g/L), Gly (~3 g/L), His (~1 g/L), Lys (~4 g/L) and Thr (~5 g/L) were added and the highest when only Ala (~37 g/L) was added [Figure 4b]. Figure 4d shows that in all SM-AA fermentations using *H. uvarum* acetic acid was detected. Besides the control, the highest acetic acid values occurred in Arg and Gln (both ~0.5 g/L) fermentations. Apart from the SM-AA Trp fermentations, which showed the highest acetic acid concentrations (~0.2 g/L), lower amounts of acetic acid were detected in all other SM-AA fermentations with *H. osmophila* than in the corresponding fermentations using *H. uvarum* [Figure 4e]. *H. osmophila* did not produce acetic acid in the SM-AA fermentations with Cys. *Z. rouxii* produced the highest amount of acetic acid when Phe was added (~0.2 g/L), even significantly exceeding the amount of acetic acid formed in the control [~0.17 g/L; Figure 4f].

**Figure 5:**
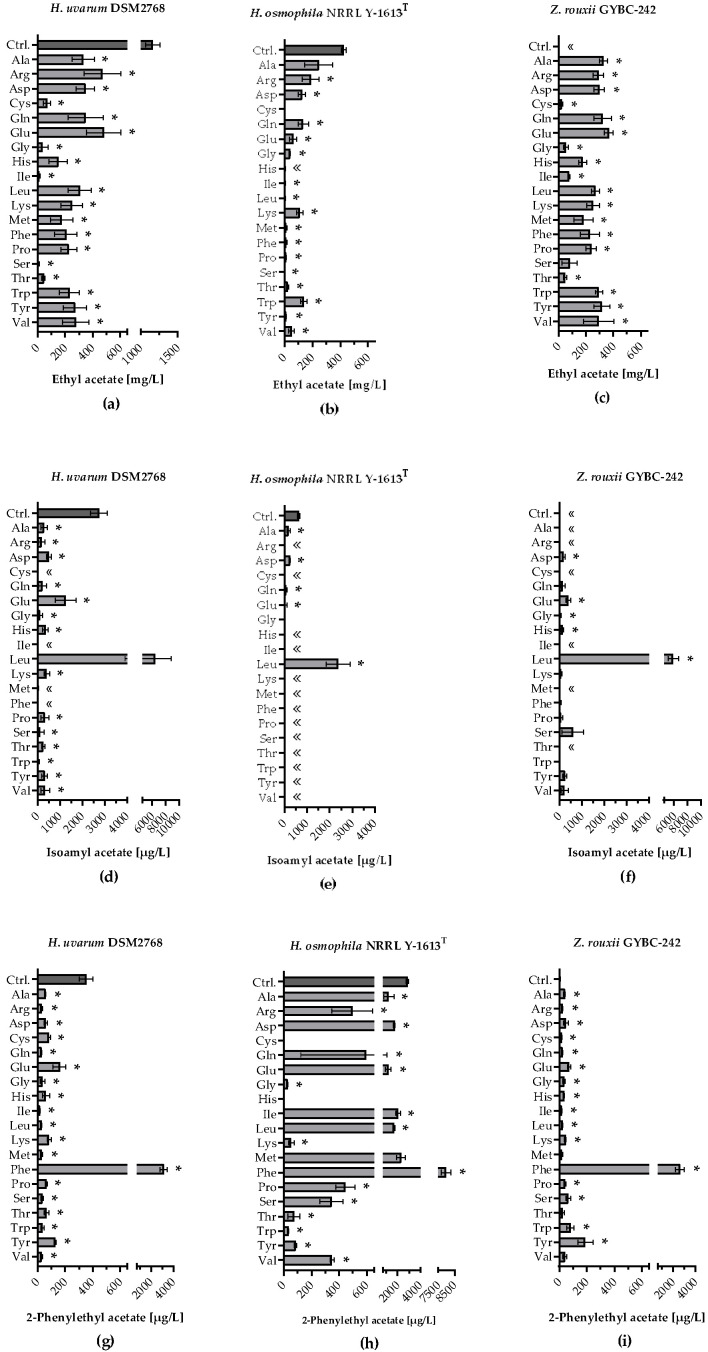
Production of acetate esters during fermentation with individually supplemented amino acids. Ethyl acetate production [mg/L] in fermentations using (**a**) *H. uvarum* DSM2768, (**b**) *H. osmophila* NRRL Y-1613T and (**c**) *Z. rouxii* GYBC-242. Isoamyl acetate production [µg/L] in fermentations using (**d**) *H. uvarum* DSM2768, (**e**) *H. osmophila* NRRL Y-1613T and (**f**) *Z. rouxii* GYBC-242. 2-Phenylethyl acetate production [µg/L] in fermentations using (**g**) *H. uvarum* DSM2768, (**h**) *H. osmophila* NRRL Y-1613T and (**i**) *Z. rouxii* GYBC-242. Fermentations using the respective yeast with SM-mix served as control (Ctrl.). Acetate esters were measured via HS-SPME-GC-MS analysis. Data are the mean of three independent experiments ± SEM, two-tailed unpaired *t* test with Welch’s correction, * *p* < 0.05 as compared to the control. Error bars indicate the standard deviation; «: not quantifiable.

Ethyl acetate, 2-phenylethyl acetate and isoamyl acetate are among the most aroma-relevant acetate esters produced during wine fermentation. In Figure 5a it can be seen that ethyl acetate was detected in all fermentations using *H. uvarum*. Besides the control (~1163 mg/L), the highest values were measured with the addition of Arg (~470 mg/L) or Glu (~478 mg/L). However, when only Ile or Ser was added, relatively small amounts of ethyl acetate were formed (Ile: ~11 mg/L; Ser: ~4 mg/L). 

*Z. rouxii* was also able to produce ethyl acetate in all SM-AA fermentations [Figure 5c]. In contrast to *H. uvarum*, however, no ethyl acetate was quantifiable in the control. The highest amount of ethyl acetate was formed when Glu was added (~365 mg/L) and the lowest when Cys was added (~22 mg/L). Many of the SM-AA fermentations using *H. osmophila* resulted in low ethyl acetate levels [below 10 mg/L; Figure 5b]. The addition of Ala yielded the highest amount of ethyl acetate (~245 mg/L). In all three yeast strains, the highest concentration of isoamyl acetate was obtained in fermentations with the addition of Leu [Figure 5d–f]. Compared to the other two yeast strains, *H. uvarum* was able to use almost all amino acids for the production of isoamyl acetate, apart from four amino acids, which were not quantifiable. Figure 5g–i shows that the addition of Phe resulted in the highest production of 2-phenylethyl acetate in all three yeast strains. Except for the SM-AA fermentations using *H. osmophila* with Cys and His and the control fermentation using *Z. rouxii*, 2-phenylethyl acetate was detected in all fermentations with the three yeast strains. In addition to the control, fermentations with *H. osmophila* with the addition of eight single amino acids, respectively, showed 2-phenylethyl acetate levels higher than 500 µg/L. In contrast, only the addition of Phe in the fermentations using *H. uvarum* and *Z. rouxii* resulted in 2-phenylethyl acetate amounts of this quantity.

**Figure 6:**
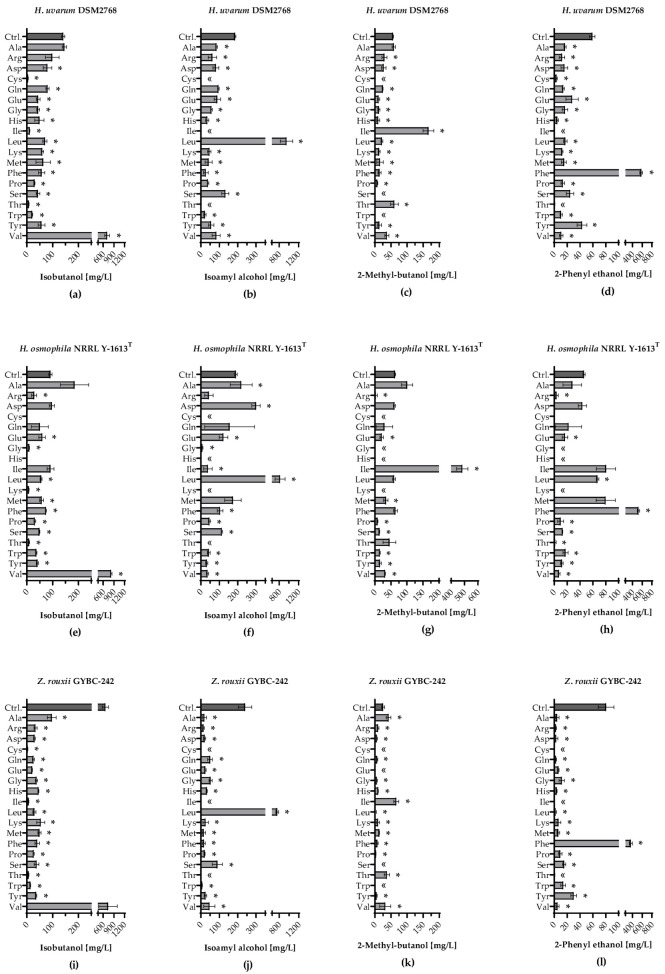
Formation of higher alcohols during fermentation with individually supplemented amino acids. Formation of isobutanol [mg/L] in fermentations using (**a**) *H. uvarum* DSM2768, (**e**) *H. osmophila* NRRL Y-1613^T^ and (**i**) *Z. rouxii* GYBC-242. Formation of isoamyl alcohol [mg/L] in fermentations using (**b**) *H. uvarum* DSM2768, (**f**) *H. osmophila* NRRL Y-1613^T^ and (**j**) *Z. rouxii* GYBC-242. Formation of 2-methyl butanol [mg/L] in fermentations using (**c**) *H. uvarum* DSM2768, (**g**) *H. osmophila* NRRL Y-1613^T^ and (**k**) *Z. rouxii* GYBC-242. Formation of 2-phenyl ethanol [mg/L] in fermentations using (**d**) *H. uvarum* DSM2768, (**h**) *H. osmophila* NRRL Y-1613^T^ and (**l**) *Z. rouxii* GYBC-242. Fermentations using the respective yeast with SM-mix served as control (Ctrl.). Higher alcohols were measured via HS-SPME-GC-MS analysis. Data are the mean of three independent experiments ± SEM, two-tailed unpaired *t* test with Welch’s correction, * *p* < 0.05 as compared to the control. Error bars indicate the standard deviation; «: not quantifiable.

Besides the acetate esters, the higher alcohols also play a major role in the aroma profile during wine fermentation. All three yeast strains showed the highest isobutanol amounts when Val was added [>700 mg/L; Figure 6a,e,i]. *H. osmophila* did not produce isobutanol when Cys or His was added. Apart from this, isobutanol was detected in all fermentations with the different yeasts. In the fermentations with the addition of Leu, *H. uvarum*, *H. osmophila* and *Z. rouxii* produced large amounts of isoamyl alcohol [<700 mg/L; Figure 6b,f,j)]. Besides Leu, the addition of Asp in fermentations using *H. osmophila* also showed significantly higher levels of isoamyl alcohol than the control. When Cys, Ile or Thr were added, isoamyl alcohol was not quantifiable in any fermentation using the various yeast strains. In Figure 6c,g,k) it can be seen that an addition of Ile led to a higher increase of 2-methyl butanol in all three yeast strains. Among the yeast strains tested, *Z. rouxii* produced significantly less 2-methyl butanol (~66 mg/L), in contrast to *H. uvarum* (~166 mg/L) and *H. osmophila* (~484 mg/L). In addition, the fermentations with *Z. rouxii* and Ala also produced significantly more 2-methyl butanol (~42 mg/L) than the control (~26 mg/L). Additional Phe led to significantly increased 2-phenyl ethanol production in all yeast strains [Figure 6d,h,l)]. A higher concentration of 2-phenyl ethanol than in the control (~46 mg/L) was also found in the single amino acid fermentations using *H. osmophila* with Leu (~67 mg/L).

The measured ethyl esters ethyl propionate and ethyl isobutyrate, as well as the acetate ester 2-methylbutyl acetate of the SM-AA fermentations are shown in Figure S2. It shows that SM-AA fermentations with Val resulted in significantly higher levels of ethyl propionate and ethyl isobutyrate in all yeast strains studied. In addition, elevated ethyl propionate levels were measured in the SM-AA Thr fermentations using all yeasts. When Ile was added, ethyl propionate was not found in fermentations using either *H. uvarum* or *Z. rouxii*. In comparison, less ethyl propionate was produced in SM-AA fermentations with *H. osmophila* with the respective nitrogen source. Besides Val, a significant increase in ethyl isobutyrate was also observed in fermentations using *H. osmophila* when Ala was added. In the SM-AA Cys fermentations, ethyl isobutyrate was not detected for any of the yeasts. The addition of Ile yielded higher concentrations of 2-methylbutyl acetate. Higher concentrations of 2-methylbutyl acetate were measured in the SM-AA fermentations with *H. uvarum* in the case of most nitrogen sources than in the respective fermentations using the other two yeasts.

Malic acid and isovaleric acid were also measured (Figure S3). Isovaleric acid could only be detected in the SM-AA Leu fermentations with all yeast strains [Figure S3d–f]. Isovaleric acid was not quantifiable in any of the SM-mix fermentations (Ctrl.). Malic acid was found in each of the culture supernatants [Figure S3a–c]. When Pro was added as the only nitrogen source, significantly less malic acid was found in fermentations of all three yeast strains. When Thr was added, however, higher malic acid values than in the SM-mix fermentations were measured in all different yeast fermentations.

The calculation of total alcohols, total acetate esters and total ethyl esters provides information about their synthesis depending on the SM-AA fermentations using the various yeast strains. Table 5 shows the total alcohols and total acetate esters, produced through the Ehrlich pathway (see also Table 1), and total ethyl esters formed in the SM-mix fermentations (Ctrl.) and SM-AA fermentations using the individual yeast strains. In the SM-mix fermentations, *Z. rouxii* formed more total alcohols than *H. uvarum* and *H. osmophila*. The latter produced the highest amounts of total acetate esters, while *H. uvarum* produced the most total ethyl esters.

Comparing the SM-AA fermentations using all three strains, *H. uvarum* formed the highest amounts of total alcohols (1.06 g/L) when Leu was added. All three strains formed high amounts of total alcohols when Leu, Phe or Val were added. In fermentations with Cys, on the other hand, only very low or no alcohols were formed. In 53% of the nitrogen sources tested in the SM-AA fermentations, *H. osmophila* produced more total alcohols than the other two yeast strains, followed by *H. uvarum* with 47%.

The highest amount of total acetate esters formed was detected in the SM-AA Phe fermentations using *H. osmophila* at 7.99 mg/L. The highest total acetate esters were measured in fermentations with Phe and Leu in all three yeast strains. Whereas the lowest concentrations were found in the fermentations with Cys. *H. uvarum* formed higher amounts of total acetate esters in 45% of the nitrogen sources tested and *H. osmophila* in 42%. *Z. rouxii* was found to be the highest total acetate ester producer in 13% of the nitrogen sources tested. *Z. rouxii* showed poorly measurable total acetate esters in the SM-mix fermentations (0.01 mg/L), whereas high amounts were formed in the SM-AA fermentations with Leu (5.92 mg/L) and Phe (2.68 mg/L).

When Val was added, all three yeast strains showed the highest total ethyl ester values, with *Z. rouxii* producing significantly more than *H. uvarum* and *H. osmophila*. The lowest amounts of measured ethyl esters were formed when Ile was added. *H. osmophila* produced more total ethyl esters in 5% of the nitrogen sources tested and *H. uvarum* in 32%. *Z. rouxii* formed more total ethyl esters than *H. uvarum* and *H. osmophila* in 63% of the SM-AA fermentations.

## 4. Discussion

From a winemaking perspective, nitrogen has a dual role, by being essential for yeast growth to complete the alcoholic fermentation and by providing the backbone structures for many important wine aroma compounds [33]. The alarming world-wide trend in increased weather temperatures directly affects the development of the grape berry leading to earlier harvest times and higher sugar content [34]. Apart from eventually leading to increased alcohol levels in the final wines, this also leads to a bigger imbalance in the sugar to nitrogen levels in the must. Also, higher temperatures and limited irrigation water are associated with lower amino acid levels in grape berries [35,36]. Furthermore, the nitrogen content can also influence the formation of volatile and non-volatile components. Lower nitrogen levels are associated with the synthesis of branched-chain fatty acids and esters in *S. cerevisiae*, while high nitrogen levels lead to the formation of medium-chain fatty esters and acetic acid, depending on the nitrogen source added [33,37]. In order to prevent the associated fermentation problems, it is important to gain more insight in the exact nitrogen requirements of the common non-*Saccharomyces* yeasts that are present in the must.

In order to investigate the fermentation performance of the different yeast strains, fermentations were carried out in SM-mix, measuring the cumulative pressure over a period of ten days. Among all yeasts tested, only *H. osmophila* was able to finish the fermentation although *S. cerevisiae* showed the steadiest pressure increase thus reaching the highest value of cumulative pressure. The fructophilic yeasts (*Z. rouxii* and *S. bacillaris*) showed similar fermentation behaviour, with a slightly lower cumulative pressure formation than in the fermentations with *H. osmophila*. However, in the SM-mix fermentations, *H. uvarum* showed by far the lowest fermentation performance. The results are in accordance to previous studies. *S. bacillaris* as well as *H. uvarum* are known to be slow fermenting yeasts, with *H. uvarum* showing even lower fermenting performances in synthetic must fermentations [38]. Whereas *H. osmophila* proved to be a good fermenter in synthetic must fermentations [39].

From our second experiment, we observed that the *H. uvarum* DSM strain, an apiculate yeast, often cited as the most abundant yeast at the onset of a fermentation, consumed the amino acids the quickest of all the yeasts in this study. Similarly, Roca-Mesa et al. [15] observed that *H. uvarum* and *S. bacillaris* utilized amino acids faster in the absence of ammonium and behave similarly in terms of amino acid uptake in anaerobic fermentations. In contrast to *H. uvarum*, *H. osmophila* had a similar amino acid utilization profile than *S. cerevisiae. H. uvarum* and *H. osmophila* belong to different lineages within the *Hanseniaspora* genus, namely the fast-evolving lineage (FEL) and the slow-evolving lineage (SEL), respectively and it has been shown, at least within the genes involved in the glycolytic pathway that members of SEL are closer in enzyme identity to *S. cerevisiae* [40]. Moreover, transcriptome data of *H. vineae* (another member of SEL) showed a similar gene upregulation pattern regarding their nitrogen utilization than that of *S. cerevisiae* which suggest these latter two yeast species share a similar nitrogen catabolism mechanism [17]. Contrary to the findings of Roca Mesa et al. [15], the two *S. bacillaris* strains and *Z. rouxii* performed similarly in terms of amino acid utilization and were significantly slower in degradation performance than *S. cerevisiae* and *H. osmophila*. Lysine was preferentially consumed by all yeast strains. This finding is consistent with previously published data [19]. Barrajón-Simancas et al. [41], however, demonstrated that different *S. cerevisiae* strains all preferred cysteine and tyrosine, but also glycine and alanine. Thirty-two hours after inoculation of the media with different yeast strains, amino acids were not detected in the fermentation supernatant, including proline, which was one of the last amino acids still to be found in the fermentation medium. Gobert et al. [42] reported that *S. bacillaris* did not consume histidine, methionine, threonine or tyrosine. Our study shows that although these amino acids were depleted after 32 h, they were among the last amino acids to be assimilated. The intermediate increase of tryptophan in the medium in fermentations with fructophilic yeasts and especially *H. osmophila* and *S. cerevisiae* was remarkable. It is known that yeasts release amino acids into the medium especially towards the end of fermentation or also in the presence of higher salt concentrations in the medium [41,43,44].

Generalising for all yeast strains investigated, lysine was most strongly preferred by all yeasts, as already described by Crépin et al. [19]. Next was a strong preference of all yeasts for Ile, Leu and Met. Besides Phe, Ser, Thr, Glu and Asp, also Ala and Val were among the preferred amino acids. Gly, Arg, His, Pro, Trp and Tyr, on the other hand, were taken up more slowly. *H. uvarum* stood out particularly in amino acid uptake. It was interesting that *H. uvarum* had already completely consumed seven of the amino acids examined within 16 h after inoculation. Most of the remaining amino acids in the fermentation supernatant were also found in significantly lower concentrations than in the fermentations with the other yeasts.

*S. cerevisiae* strains are known for their strong glucophilic properties [45]. The results of this study also show the preference for glucose by *H. uvarum* and especially *H. osmophila*. Among these three glucophilic yeasts, *S. cerevisiae* showed the strongest glucophilic properties, followed by *H. osmophila. H. uvarum*, showing to be moderately glucophilic, absorbed proportionally more fructose compared to the other two yeast strains. In contrast, both *Z. rouxii* and the two *S. bacillaris* strains displayed a strong fructophilic phenotype [46,47]. Of the three, *Z. rouxii* consumed proportionally more fructose than glucose. 

For further analyses of the single amino acid fermentations and the synthesis of aroma relevant compounds, we selected *H. uvarum*, *H. osmophila* and *Z. rouxii. H. uvarum* plays a major role in spontaneous fermentations as it is abundant in the grape microbiome. Moreover, *H. uvarum* stood out in amino acid uptake and sugar degradation. Due to the similarities to *S. cerevisiae* in terms of sugar degradation and amino acid degradation, we decided to investigate amino acid metabolism and the resulting alcohols and esters in fermentations with *H. osmophila* in more detail. *H. uvarum* and *H. osmophila* are two different yeasts of one yeast genus, which also correspond to different lineages (FEL and SEL), that can be compared with each other. Since all fructophilic yeast strains tested, showed a similar behaviour regarding both the amino acid uptake and the sugar degradation, *Z. rouxii* was chosen for further experiments as *Z. rouxii* revealed slightly stronger fructophilic properties.

The Ehrlich pathway-mediated catabolism of the branched chain amino acids (Val, Leu and Ile), the aromatic amino acids (Phe, Tyr and Trp) and the sulphur-containing amino acid Met produces compounds that are the main yeast-derived aroma contributors in wine and other alcoholic beverages [10,48,49]. As expected, feeding leucine led to an increase in isoamyl alcohol and isoamyl acetate in all three yeast strains studied. Isoleucine was converted to 2-methyl butanol and 2-methylbutyl acetate as predicted. Valine is converted to isobutanol and isobutyl acetate in the Ehrlich pathway leading to a pineapple and banana-like aroma. The SM-AA fermentations using the various yeast strains with valine addition showed the increased production of isobutanol, ethyl isobutyrate as well as ethyl propionate. α-Keto isovalerate, which is formed from valine in a transaminase reaction, can not only be converted to isobutanol or isobutyrate via 2-methylpropanal, but can also be converted into α-keto-isocaproate and then introduced into the leucine catabolism [7]. There, it is converted into isoamyl alcohol or isoamyl acetate. Thus, in the SM-AA Val fermentations using the three yeast strains, significant amounts of isoamyl alcohol and, in the case of *H. uvarum*, isoamyl acetate were also detected. Compared to the other two yeasts, *H. uvarum* was able to form isoamyl acetate from almost all amino acids tested in the SM-AA fermentations. This was also observed previously for *S. cerevisiae* [50]. In contrast to our studies, in addition to leucine, it was also isoleucine that led to a higher production of isoamyl acetate and isoamyl alcohol in fermentations using *S. cerevisiae*. Threonine is first converted to α-ketobutyrate and then further to either 1-propanol or 1-butanol. α-ketobutyrate can also be converted to alpha-keto-3-methylvalerate, which is then introduced into the isoleucine catabolism and further converted to 2-methyl-butanol [7]. This can be seen in this experiment from the amount of 2-methyl butanol formed in the SM-AA Thr fermentations using the three yeast strains. Of the three strains, *H. uvarum* produced the largest amounts of both 2-methyl butanol in the SM-AA Thr and isoamyl alcohol in the SM-AA Val fermentations. The aromatic amino acid phenylalanine also led to the production of large amounts of 2-phenyl ethanol and 2-phenylethyl acetate, which are known for producing the typical rose-like aroma. Even though methionine is known to be converted into methionol and 3-(methylthio) propanoate, within the Ehrlich pathway, it also had a general influence on the formation of alcohols and esters. This is particularly seen in fermentations inoculated with *H. osmophila*. In addition to phenylalanine, methionine as well as isoleucine appeared to have a particularly strong influence on the formation of 2-phenylethyl acetate and the corresponding alcohol. This is in contrast to previously published studies on fermentations with *S. cerevisiae* [50]. Fairbairn et al. [50] showed that only the addition of phenylalanine led to the production of 2-phenylethyl acetate.

The SM-AA fermentations with leucine led not only to the strong production of isoamyl alcohol in *H. osmophila*, but also to the formation of 2-methyl butanol and 2-phenyl ethanol. This discovery was also made by Espinosa Vidal et al. [51] in fermentations using *S. cerevisiae* with an oversupply of leucine, as the only nitrogen and carbon source. They proposed that these substances are biosynthesised *de novo* as outflows of valine, isoleucine and phenylalanine in *S. cerevisiae*. We did not observe this in the SM-AA Leu fermentations using *H. uvarum* and *Z. rouxii*. This finding supports our assumption that *H. osmophila* and *S. cerevisiae* have a similar amino acid metabolism, as both yeasts show a similar amino acid degradation profile in the SM-mix fermentations. *H. osmophila* showed no fermenting activity in the SM-AA fermentations with only lysine or histidine as amino acid source, conforming to its reported similarities with *S. cerevisiae* [52]. 

It is noticeable that *Z. rouxii* formed only a few (quantifiable) acetate- and ethyl esters under the fermentation conditions of the SM-mix fermentations. In the SM-AA fermentations, on the other hand, compared to the other yeast strains, it produced more total ethyl esters in most of the nitrogen sources tested. *H. uvarum* formed the highest amounts of total ethyl esters in the SM-mix fermentations. Interestingly, *Z. rouxii* was able to form high amounts of total acetate esters in the SM-AA fermentations, especially with leucine and phenylalanine, although this was not observed in the SM-mix fermentations. This suggests that there is some kind of inhibition of acetate ester production in the presence of all amino acids. Even though *H. osmophila* formed the highest amounts of total acetate esters in the SM-mix fermentations and also in SM-AA fermentations (with Phe added), *H. uvarum* formed more acetate esters in the fermentations in most of the nitrogen sources tested.

As a typical feature of *H. uvarum*, it produced the highest level of ethyl acetate, which is by far the most abundant ester found in alcoholic fermentations. In contrast to *H. osmophila*, which displayed relatively weak ester formation, ethyl acetate was detected in every SM-AA fermentation using *H. uvarum* and *Z. rouxii*. The amino acids Ala, Arg, Asp, Gln and Glu, which are not primarily associated with the production of aromatic acetate esters in the Ehrlich pathway, appear to have an important role not only in the formation of ethyl acetate, but also in total acetate ester formation. Even though *H. osmophila* is rather weak in terms of total ester production, in addition to SM-AA Phe fermentations, SM-AA fermentations with seven other amino acids reached 2-phenylethyl acetate values of over 1000 µg/L. While *H. osmophila* uses many amino acids for a strong 2-phenylethyl acetate formation, no 2-phenylethyl acetate was detected in the SM-AA fermentations with cysteine and histidine. 

Regarding the SM-mix fermentations, *Z. rouxii* showed an increased formation of total alcohols. In the SM-AA fermentations, however, they always produced fewer total alcohols than the *Hanseniaspora* strains. Although the highest level of total alcohol was detected in the SM-AA fermentations with *H. uvarum* (with the addition of leucine), *H. osmophila* produced the highest levels of total alcohols in most fermentations with different nitrogen sources. In addition to the amino acids known to be relevant for the Ehrlich pathway, arginine and aspartic acid also appeared to have an influence on total alcohol production, especially by *H. osmophila*.

Our data show that leucine and phenylalanine have a significant influence on total alcohol and total acetate ester production in all yeast strains tested. Valine leads to an increase in total alcohol and total ethyl ester synthesis. Comparing the SM-AA fermentations with each other, it is noticeable that cysteine leads to a low yield of total alcohols, total acetate esters and total ethyl esters in all yeast strains. This could be explained by the inhibited growth of yeasts in SM-AA fermentations spiked with cysteine, as already reported by Su et al. [16]. In the SM-AA fermentations with *H. osmophila*, histidine also leads to inhibited growth and the absence of total alcohols and total esters in the fermentation supernatant. Su et al. [16] showed that the growth of *S. cerevisiae* was also inhibited by the addition of histidine as the sole nitrogen source. Due to the similarity in amino acid uptake and similar behaviour in amino acid-related aroma synthesis of *H. osmophila* and *S. cerevisiae*, this is according to the observations of Su et al. [16].

*H. uvarum* produced the highest amounts of acetic acid in both the SM-mix and SM-AA fermentations. In contrast to *H. osmophila* and *Z. rouxii*, acetic acid levels in *H. uvarum* were only very low (<0.1 g/L) in the SM-AA fermentations with glycine and threonine. Interestingly, SM-AA fermentations with proline, which led to significant amounts of ethanol in all yeast strains, resulted in a very low amount of acetic acid in fermentations using the various yeast strains. The presence of oxygen enables its degradation and makes it “yeast-available” [53]. This is also evident in the results of this study. In the fermentations with proline, the yeasts studied showed an increased production of total esters.

In a comparison of all amino acids as the only nitrogen source of the yeasts tested, leucine and phenylalanine are the most desirable in conferring general benefits to wine fermentations. Not only are they particularly strong in the production of aromatic components and contribute to the quality of the aroma profile of alcoholic end products, but they also enable the yeasts to run a strong fermentation when they are the only source of nitrogen. Isoleucine is also important in terms of flavour diversity, but leads to a weaker fermentation progress if no other nitrogen sources are available.

## 5. Conclusions

In conclusion, nitrogen requirements of non-*Saccharomyces* yeasts, as well as their utilization, are species dependent. Even yeasts belonging to the same genus may have different nitrogen uptake and utilization profiles. Also, glucophilic and fructophilic yeasts can produce similar aroma profiles as a function of different nitrogen sources. This work highlights information about the amino acid preferences of selected NSY and how it influences the production of aroma compounds relevant in a winemaking context.

## Figures and Tables

**Table 2:** Composition of the synthetic must base without the added amino acids. All chemicals were obtained from Merck (Darmstadt, Germany).

Component	Concentration
**Sugars**	**[g/L]**
Glucose	100
Fructose	100
**Acids**	**[g/L]**
DL-Malic acid	5
Citric Acid	0.5
L-(+)-Tartaric acid	3
**Minerals**	**[mg/L]**
KH_2_PO_4_	750
K_2_SO_4_	500
MgSO_4_	250
CaCl_2_	160
NaCl	200
**Trace elements**	**[mg/L]**
MnSO_4_	4
ZnSO_4_	4
CuSO_4_	1
KI	1
CoCl_2_	0.4
H_3_BO_3_	1
(NH_4_)_6_Mo_7_O_24_	1
**Vitamins**	**[mg/L]**
Myo-inositol	20
Calcium pantothenate	1.5
Nicotinic acid	2
Thiamine hydrochloride	0.25
Pyridoxine hydrochloride	0.25
Biotin	0.003
**Phytosterol solution for 100 L of SM**	
β-Sitosterol	500 mg
Tween 80	16.7 mL
Ethanol pure	16.7 mL

**Table 3:** Amino acid and ammonium concentrations of the synthetic must with amino acid mix (SM-mix) and when used individually in the single amino acid fermentations (SM-AA).

Amino Acid	SM-mix [mg/L] *	SM-AA [mg/L] *
L-alanine	48	891
L-arginine	123	581
L-asparagine	0	661
L-aspartic acid	15	1331
L-cysteine	4	1212
L-glutamine	40	1471
L-glutamic acid	166	731
Glycine	6	751
L-histidine	11	1552
L-isoleucine	11	1312
L-leucine	16	1312
L-lysine	6	731
L-methionine	10	1492
L-phenylalanine	13	1652
L-proline	202	1151
L-serine	26	1051
L-threonine	25	1191
L-tryptophan	59	2042
L-tyrosine	6	1812
L-valine	15	1172
NH_4_Cl	151	535

* The respective concentrations were taken from Su et al. [16].

**Table 4:** Analysis of glucose, fructose and total sugar in yeast fermentations with SM-mix ^a, b^.

Strain	Glucose [g/L]	Fructose [g/L]	Total Sugar [g/L]	Glucose * [%]	Fructose * [%]
*H. uvarum* DSM2768	52.46 ± 0.74	62.67 ± 1.70	115.13 ± 2.45	45.57	54.43
*H. osmophila* NRRL Y-1613^T^	33.29 ± 0.79	55.79 ± 0.71	89.08 ± 1.36	37.37	62.63
*S. cerevisiae* GHM	30.70 ± 0.80	68.83 ± 0.90	99.54 ± 1.70	30.84	69.15
*Z. rouxii* GYBC-242	98.18 ± 1.31	20.09 ± 0.86	118.26 ± 2.17	83.02	16.99
*S. bacillaris* GYBC-240	95.06 ± 0.84	19.84 ± 0.68	114.90 ± 0.17	82.73	17.27
*S. bacillaris* GYBC-241	97.18 ± 0.94	22.46 ± 1.00	119.65 ± 0.49	81.22	18.77
SM ^c^	101.24 ± 2.17	102.06 ± 2.20	203.30 ± 4.36	49.80	50.20

^a^ Samples were analysed via HPLC three days after inoculation. ^b^ Data are the mean of three independent experiments ± SD. ^c^ Synthetic must (SM) was analysed to measure the initial concentration of sugars. * The percentage of each sugar remaining in the supernatant.

**Table 5:** Overview of the production of total alcohols, total acetate esters and total ethyl esters in SM-mix (Ctrl.) and SM-AA fermentations using *H. uvarum* DSM2768 (*H*.*u*.), *H. osmophila* NRRL Y-1613^T^ (*H*.*o*.) and *Z. rouxii* GYBC-242 (*Z*.*r*.). Darker colours indicate higher concentrations within the total alcohols, total acetate esters and total ethyl esters, lighter colours indicate lower concentrations within the respective group.

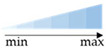									
	Total Alcohols ^a^ [g/L]			Total Acetate Esters ^b^ [mg/L]			Total Ethyl Esters ^c^ [mg/L]		
	*H*.*u*.	*H*.*o*.	*Z*.*r*.	*H*.*u*.	*H*.*o*.	*Z*.*r*.	*H*.*u*.	*H*.*o*.	*Z*.*r*.
Ctrl.	0.44	0.39	1.01	3.47	3.58	0.01	1.88	0.78	0.28
Ala	0.31	0.53	0.16	0.43	1.45	0.10	0.50	0.26	0.43
Arg	0.20	0.08	0.06	0.26	0.50	0.04	0.36	0.07	0.34
Asp	0.20	0.50	0.06	0.62	1.96	0.26	0.44	0.25	0.40
Cys	0.01	0.00	0.00	0.08	0.00	0.02	0.08	0.00	0.03
Gln	0.22	0.26	0.08	0.27	0.66	0.19	0.45	0.15	0.46
Glu	0.17	0.22	0.05	1.47	1.21	0.49	0.45	0.03	0.37
Gly	0.13	0.02	0.11	0.13	0.03	0.09	0.06	0.00	0.09
His	0.10	0.00	0.09	0.41	0.00	0.20	0.17	0.00	0.25
Ile	0.18	0.69	0.07	0.23	2.57	0.17	0.00	0.01	0.00
Leu	1.06	1.00	0.79	6.40	4.08	5.92	0.38	0.03	0.34
Lys	0.13	0.01	0.09	0.51	0.05	0.15	0.22	0.04	0.24
Met	0.14	0.35	0.08	0.06	2.30	0.03	0.14	0.05	0.28
Phe	0.68	0.76	0.43	3.15	7.99	2.68	0.18	0.08	0.24
Pro	0.09	0.09	0.06	0.41	0.44	0.15	0.26	0.00	0.34
Ser	0.20	0.19	0.14	0.13	0.35	0.68	0.00	0.00	0.06
Thr	0.07	0.05	0.04	0.40	0.12	0.11	0.52	0.49	0.77
Trp	0.05	0.11	0.03	0.06	0.04	0.10	0.19	0.07	0.28
Tyr	0.17	0.10	0.10	0.47	0.09	0.47	0.26	0.00	0.40
Val	0.84	0.90	0.82	0.41	0.36	0.34	5.25	3.30	13.68

^a^ Total alcohol produced through Ehrlich pathway including isobutanol, isoamyl alcohol, 2-methyl butanol and 2-phenyl ethanol. ^b^ Total acetate esters produced through Ehrlich pathway including isoamyl acetate, 2-methylbutyl acetate and 2-phenylethyl acetate. ^c^ Total ethyl esters including ethyl propionate and ethyl isobutyrate.

## Data Availability

Not applicable.

## Supplementary Materials

The following supporting information can be downloaded at: https://www.mdpi.com/article/10.3390/microorganisms11010014/s1, Figure S1: Amino acid concentration (%) present in the medium at different time points of the alcoholic fermentation in SM-mix. The initial concentration of each amino acid is expressed as 100%. It should be noted that the values of Glu also contained Gln and Asp also contained Asn. Cys could not be measured.; Figure S2: Formation of ethyl- and acetate esters during fermentation with individually supplemented amino acids. Formation of ethyl propionate [µg/L] in fermentations using (a) H. uvarum DSM2768, (b) H. osmophila NRRL Y-1613T and (c) Z. rouxii GYBC-242. Formation of ethyl isobutyrate [µg/L] in fermentations using (d) H. uvarum DSM2768, (e) H. osmophila NRRL Y-1613T and (f) Z. rouxii GYBC-242. Formation of 2-methylbutyl acetate [µg/L] in fermentations using (g) H. uvarum DSM2768, (h) H. osmophila NRRL Y-1613T and (i) Z. rouxii GYBC-242. Fermentations using the respective yeast with SM-mix served as control (Ctrl.). Higher alcohols were measured via HS-SPME-GC-MS analysis. Data are the mean of three independent experiments ± SEM, two-tailed unpaired t test with Welch’s correction, * *p* < 0.05 as compared to the control. Error bars indicate the standard deviation; «: not quantifiable.; Figure S3: Acid production during fermentation with individually supplemented amino acids. Malic acid production [g/L] in fermentations using (a) H. uvarum DSM2768, (b) H. osmophila NRRL Y-1613T and (c) Z. rouxii GYBC-242. Isovaleric acid production [µg/L] in fermentations using (d) H. uvarum DSM2768, (e) H. osmophila NRRL Y-1613T and (f) Z. rouxii GYBC-242. Fermentations using the respective yeast with SM-mix served as control (Ctrl.). Higher alcohols were measured via HS-SPME-GC-MS analysis. Data are the mean of three independent experiments ± SEM, two-tailed unpaired t test with Welch’s correction, * *p* < 0.05 as compared to the control. Error bars indicate the standard deviation; «: not quantifiable.
