# Supplementary material for: Synthesis of Aroma Compounds as a Function of Different Nitrogen Sources in Fermentations Using Non-Saccharomyces Wine Yeasts
Source: Microorganisms. 2022 Dec 21;11(1):14. doi: 10.3390/microorganisms11010014 (PMC9861872; doi:10.3390/microorganisms11010014)
Supplement: Supplementary file 1 [file microorganisms-11-00014-s001.zip › microorganisms-2085387-supplementary.pdf]

microorganisms

MDPI

Supplementary Materials:

Strain	Time [h]	Ala	Arg	Asp	Glu	Gly	His	Ile	Leu	Lys	Met	Phe	Pro	Ser	Thr	Trp	Tyr	Val
H. uvarum	0	100	100	100	100	100	100	100	100	100	100	100	100	100	100	100	100	100
DSM2768	8	94	96	95	92	95	94	90	91	87	88	101	96	96	98	93	94	92
	16	46	77	34	3	77	43	0	0	0	0	43	90	15	5	56	58	0
	24	0	11	0	0	0	0	0	0	0	0	0	10	1	0	0	0	0
	32	1	0	1	0	2	0	0	0	5	0	0	0	1	0	0	0	0
H. osmophila	0	100	100	100	100	100	100	100	100	100	100	100	100	100	100	100	100	100
NRRL Y-1613T	8	88	94	87	88	88	88	87	87	88	83	92	91	91	92	85	94	88
	16	75	83	31	61	90	87	70	50	0	43	64	93	73	76	122	72	74
	24	7	0	2	0	57	22	0	0	0	0	0	97	5	6	10	22	3
	32	1	0	0	0	4	0	0	0	2	0	0	0	0	0	0	0	0
S. cerevisiae	0	100	100	100	100	100	100	100	100	100	100	100	100	100	100	100	100	100
GHM	8	88	90	88	89	89	88	87	87	87	84	99	87	92	93	85	93	88
	16	91	89	71	82	95	74	65	52	0	57	71	92	75	60	123	82	80
	24	0	1	0	0	22	0	0	0	0	0	0	83	1	0	9	4	0
	32	0	0	0	0	1	0	0	0	2	0	0	0	0	0	0	0	0
Z. rouxii	0	100	100	100	100	100	100	100	100	100	100	100	100	100	100	100	100	100
GYBC-242	8	93	93	93	93	96	92	93	92	95	89	97	93	93	96	87	96	93
	16	91	86	100	95	93	105	82	73	46	74	86	97	90	97	110	76	83
	24	56	47	84	82	101	91	48	33	2	24	48	68	69	85	56	97	55
	32	1	0	0	0	2	0	0	0	0	0	0	0	0	0	0	0	0
S. bacillaris	0	100	100	100	100	100	100	100	100	100	100	100	100	100	100	100	100	100
GYBC-240	8	91	91	91	91	93	90	89	88	90	86	95	92	93	95	83	92	91
	16	90	82	100	84	101	111	76	63	25	61	81	97	87	96	100	95	81
	24	14	2	54	46	68	36	10	1	0	1	3	33	22	46	23	51	11
	32	0	0	0	0	0	0	0	0	0	0	0	0	0	0	0	0	0
S. bacillaris	0	100	100	100	100	100	100	100	100	100	100	100	100	100	100	100	100	100
GYBC-241	8	90	91	91	90	93	90	89	90	92	86	95	91	93	95	85	102	91
	16	95	88	103	96	99	107	87	78	50	77	92	103	87	97	101	95	91
	24	63	53	88	86	106	97	56	44	0	30	58	73	74	89	58	108	67
	32	0	0	0	0	0	0	0	0	1	0	0	0	0	0	0	0	0

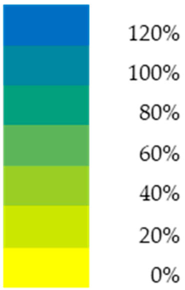

Figure S1. Amino acid concentration (%) present in the medium at different time points of the alcoholic fermentation in SM-mix. The initial concentration of each amino acid is expressed as 100%. It should be noted that the values of Glu also contained Gln and Asp also contained Asn. Cys could not be measured.

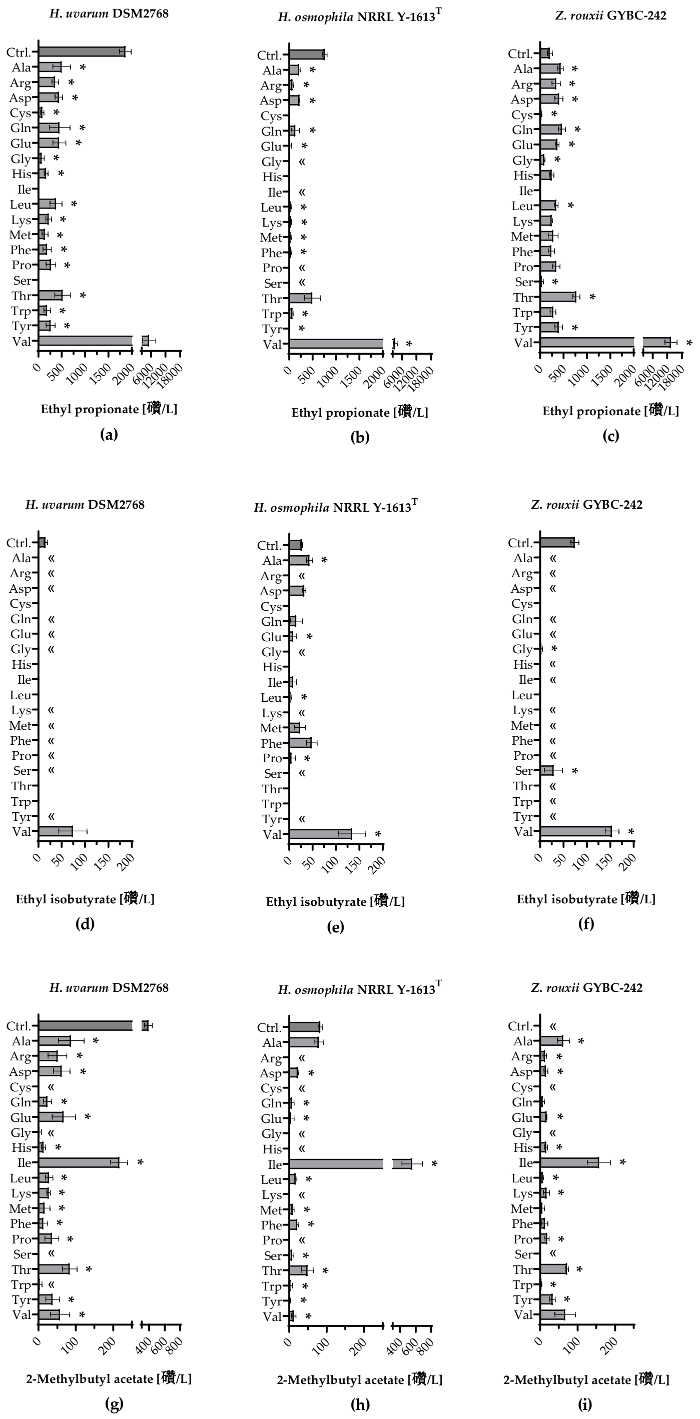

Figure S2. Formation of ethyl- and acetate esters during fermentation with individually supplemented amino acids. Formation of ethyl propionate [µg/L] in fermentations using (**a**) *H. uvarum* DSM2768, (**b**) *H. osmophila* NRRL Y-1613^T and (**c**) *Z. rouxii* GYBC-242. Formation of ethyl isobutyrate [µg/L] in fermentations using (**d**) *H. uvarum* DSM2768, (**e**) *H. osmophila* NRRL Y-1613^T and (**f**) *Z. rouxii* GYBC-242. Formation of 2-methylbutyl acetate [µg/L] in fermentations using (**g**) *H. uvarum* DSM2768, (**h**) *H. osmophila* NRRL Y-1613^T and (**i**) *Z. rouxii* GYBC-242. Fermentations using the respective yeast with SM-mix served as control (Ctrl.). Higher alcohols were measured via HS-SPME-GC-MS analysis. Data are the mean of three independent experiments ± SEM, two-tailed unpaired t test with Welch´s correction, * $p < 0.05$ as compared to the control. Error bars indicate the standard deviation; «: not quantifiable.

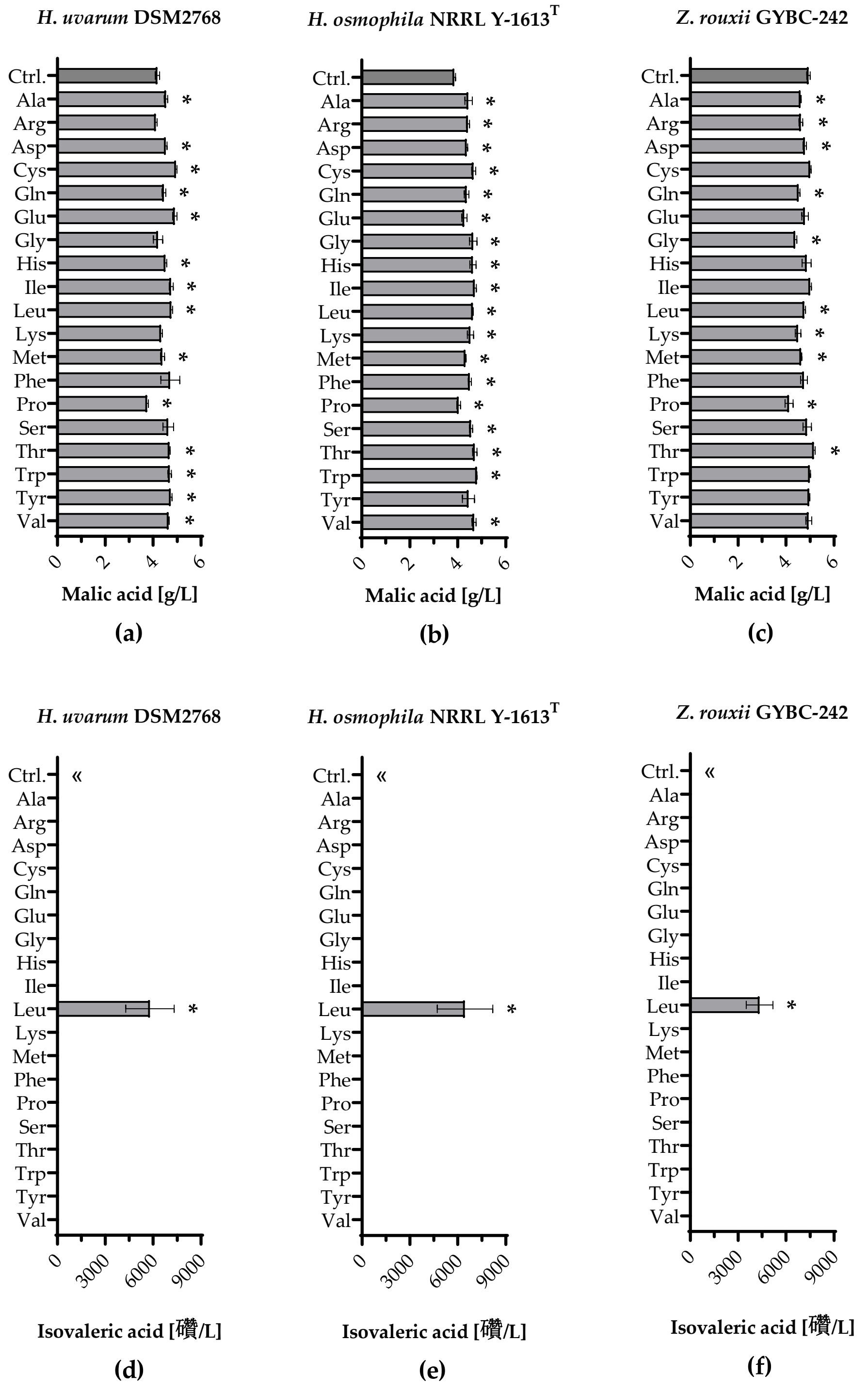

Figure S3. Acid production during fermentation with individually supplemented amino acids. Malic acid production [g/L] in fermentations using (**a**) *H. uvarum* DSM2768, (**b**) *H. osmophila* NRRL Y-1613^{T} and (**c**) *Z. rouxii* GYBC-242. Isovaleric acid production [µg/L] in fermentations using (**d**) *H. uvarum* DSM2768, (**e**) *H. osmophila* NRRL Y-1613^{T} and (**f**) *Z. rouxii* GYBC-242. Fermentations using the respective yeast with SM-mix served as control (Ctrl.). Higher alcohols were measured via HS-SPME-GC-MS analysis. Data are the mean of three independent experiments ± SEM, two-tailed unpaired t test with Welch´s correction, * $p < 0.05$ as compared to the control. Error bars indicate the standard deviation; «: not quantifiable.
